# Gene Expression Profiling of Histiocytic Sarcomas in a Canine Model: The Predisposed Flatcoated Retriever Dog

**DOI:** 10.1371/journal.pone.0071094

**Published:** 2013-08-01

**Authors:** Kim M. Boerkamp, Marieke van der Kooij, Frank G. van Steenbeek, Monique E. van Wolferen, Marian J. A. Groot Koerkamp, Dik van Leenen, Guy C. M. Grinwis, Louis C. Penning, Erik A. C. Wiemer, Gerard R. Rutteman

**Affiliations:** 1 Faculty of Veterinary Medicine, Department of Clinical Sciences Companion Animals, Utrecht University, Utrecht, The Netherlands; 2 Molecular Cancer Research, University Medical Centre Utrecht, Utrecht, The Netherlands; 3 Faculty of Veterinary Medicine, Department of Pathobiology, Utrecht University, Utrecht, The Netherlands; 4 Dept. of Medical Oncology, Daniel den Hoed Cancer Center, Erasmus University Medical Center, Rotterdam, The Netherlands; University of Texas MD Anderson Cancer Center, United States of America

## Abstract

**Background:**

The determination of altered expression of genes in specific tumor types and their effect upon cellular processes may create insight in tumorigenesis and help to design better treatments. The Flatcoated retriever is a dog breed with an exceptionally high incidence of histiocytic sarcomas. The breed develops two distinct entities of histiocytic neoplasia, a soft tissue form and a visceral form. Gene expression studies of these tumors have value for comparable human diseases such as histiocytic/dendritic cell sarcoma for which knowledge is difficult to accrue due to their rare occurrence. In addition, such studies may help in the search for genetic aberrations underlying the genetic predisposition in this dog breed.

**Methods:**

Microarray analysis and pathway analyses were performed on fresh-frozen tissues obtained from Flatcoated retrievers with localized, soft tissue histiocytic sarcomas (STHS) and disseminated, visceral histiocytic sarcomas (VHS) and on normal canine spleens from various breeds. Expression differences of nine genes were validated with quantitative real-time PCR (qPCR) analyses.

**Results:**

QPCR analyses identified the significantly altered expression of nine genes; *PPBP, SpiC, VCAM1, ENPEP, ITGAD* (down-regulated), and *GTSF1, Col3a1, CD90* and *LUM* (up-regulated) in the comparison of both the soft tissue and the visceral form with healthy spleen. DAVID pathway analyses revealed 24 pathways that were significantly involved in the development of HS in general, most of which were involved in the DNA repair and replication process.

**Conclusions:**

This study identified altered expression of nine genes not yet implicated in histiocytic sarcoma manifestations in the dog nor in comparable human histiocytic/dendritic sarcomas. Exploration of the downside effect of canine inbreeding strategies for the study of similar sarcomas in humans might also lead to the identification of genes related to these rare malignancies in the human.

## Introduction

Fundamental research on rare human diseases is not only hampered by minimal grant supply; lack of sufficient sample numbers is of equal disadvantage. One way to overcome this last catch, is to investigate other species in which a similar disease occurs at a much higher frequency, like in specific dog breeds. In dogs, a downside of selection for breeding purposes is the occurrence of a very large number of breed-specific hereditary diseases (http://omia.angis.org.au/home/). Rare human diseases, such as histiocytic malignancies, might therefore be common in specific dog breeds [1].

The Flatcoated retriever (FCR) breed has a strongly increased risk for histiocytic sarcoma (HS) development. In the UK, it is likely to account for about 36% of all malignant neoplasms diagnosed in this breed [2], [3].

Canine histiocytic malignant disorders were as such first described in the late 1970s [4]. Included in the name ‘histiocytic sarcoma’, which was given to the complex of malignant histiocytic disorders [5], there is a range of malignant tumors derived from CD34-committed stem cell precursors that may develop into dendritic cells (DC) such as Langerhans cells, interstitial DC and macrophages [6], [7]. In addition to morphological features, most histiocytic sarcomas can be recognized by positive immunostaining for the cell surface marker CD18 [6]–[8]. Canine HS has resemblance to the rare and often lethal human histiocytic malignancies, including dendritic cell and histiocytic sarcomas and disseminated Langerhans cell histiocytosis (LCH) [9]–[12]. Histiocytic sarcomas in dogs almost inevitably metastasize to various organs [10] and have a very poor prognosis [2], [10], [11]. A median survival of four months has been reported in the FCR [3].

In dogs, there are several clinical manifestations of HS. One common form is the localized, soft tissue histiocytic sarcoma (STHS), which manifests itself as a tumor arising in the deeply seated soft tissues of limbs often in association with joints [2], . A second common form is manifested in internal organs and often multifocal and named disseminated, visceral histiocytic sarcoma (VHS), with neoplastic changes that can be found in either spleen, liver, lung and/or bone marrow [6].

Past research using Comparative Genomic Hybridization has already shown various aberrations in HS in the Bernese Mountain Dog, another breed predisposed to histiocytic malignancies, with cases showing numerous shared Copy Number Alterations (CNAs) both gains and losses, throughout the genome. These included deletions of the tumor suppressor genes *CDKN2A/B, RB1* and *PTEN* [10]. Furthermore, an associated constitutional haplotype in a locus near to the highly cited tumor suppressor locus *MTAP*-*CDKN2A* has recently been identified in this breed [1]. Another study concluded that deregulation of the expression of the glycation end products (Receptor for Advanced Glycation Endproducts; *RAGE*) and the high mobility group box1 protein (*HMGB1*) potentially have a major effect on the progression of malignant histiocytic disorders [14].

cDNA microarrays have become powerful tools in the study of gene expression which has enabled improved classification of various naturally occurring cancers [15], [16] and have, once the canine genome sequence became available [17] already proven their value in the research of various canine sarcomas [18]–[20] but not yet in HS. Thus, we examined shared genetic functional aberrations of HS by comparing both forms of HS with normal tissue, for which spleen was chosen.

The study of spontaneously occurring tumors in the dog, a species which has a genetically stronger relationship to the human than mice [21], [22] can enrich the knowledge of rare human cancers, and lead to more insight in the pathogenesis of the disease and facilitate the identification of therapeutic targets valuable for dog and human [1], [11], [18], [20], [21]. The outcome of this study provides evidence of the existence of common differences in gene activity between HS and normal spleen.

## Materials and Methods

The experimental protocol (ID 2007.III.08.110) was peer-reviewed by the scientific committee of the Department of Animals in Science & Society, Utrecht University, The Netherlands, and approved by the Animal Experiments Committee of the Academic Biomedical Centre, Utrecht, The Netherlands. The Animal Experiments Committee based its decision on 'De Wet op de Dierproeven' (The Dutch 'Experiments on Animals Act', 1996) and on the 'Dierproevenbesluit' (the Dutch 'animal experiments decree', 1996). Both documents are available online at http://wetten.overheid.nl.

### Case Recruitment and Histopathological Evaluation

All tumor samples were confirmed as being spontaneously occurring histiocytic malignancies and were obtained from family-owned FCR with informed owner consent. All tumor material used originated from the Dutch FCR, that had not received radiotherapeutical or cytostatic treatment. Tumors were obtained under sterile conditions, either as part of a routine diagnostic or therapeutic surgical procedure, or immediately following euthanasia. Directly after excision, samples were snap frozen in liquid nitrogen, or alternatively by primary preservation in RNA-later, in both instances followed by storage at minus 70°C. Tumor samples collected adjacent to the site of the frozen or RNA-later preserved samples were fixed in 10% neutral buffered formalin and routinely processed for histological examination.

At the time of surgery or necropsy, the evident anatomical location of all tumors was recorded for each individual and categorized as either VHS, if a tumor was present in internal organs (n = 7) or STHS, if the tumor was localized in a limb only without identifiable metastases (n = 6) [8].

Histological specimens were classified by a board-certified veterinary pathologist (GCMG) according to the recommendations and classification scheme defined by Affolter and Moore [6]. In all cases immunohistochemical staining with antibodies against CD18 protein (the common subset of β2 adhesion integrins, expressed in histiocytes, dendritic cells (DC), lymphocytes, and polymorphonuclear leukocytes [23]) were used to confirm the suspected histiocytic origin [8]. Results of this staining were divided in two categories: negative or positive. If the differential diagnosis based upon morphology included the potential origin of other malignant round cell tumors (malignant lymphoma, mastocytoma, melanoma, myeloma) appropriate immunostaining to examine such potential histogentic origin was performed and had to be negative. All tumor samples selected for the genetic study contained over 50% tumor cells as assessed in histological sections of biopsies of adjacent tissue. Patient details are listed in Table 1.

**Table 1:** Patient details.

Name	Sex	Pathology	AO (yrs)	Site(s)	Microarray/PCR
D1, TJ	MN	STHS	7.7	shoulder	Y/Y
D3, UH	FN	STHS	8	shoulder	Y/Y
D4, BS	MN	STHS	6.5	elbow	Y/Y
D5, BaS	M	STHS	7.6	knee	Y/Y
D6, TV	MN	STHS	8.1	elbow	Y/Y
D7, DV	MN	STHS	11	shoulder	Y/Y
DX, YM	MN	STHS	9.7	shoulder	N/Y
D2, BE	M	VHS	9.4	liver/spleen/**lnn abd**	Y/Y
D8, DW	M	VHS	9.5	spleen/**lnn abd**	Y/N
D9, JV	FN	VHS	8.9	lung/**lnn mediast**	Y/Y
D11, BT	F	VHS	8.5	**lung**	Y/Y
D12, AG	M	VHS	7.3	**lung**/spleen/kidney	Y/Y
D13, TR	FN	VHS	7.9	**lung**	Y/Y
D14, SG	F	VHS	4.1	lung/**lnn mediast**	Y/Y
DX, SC	MG	VHS	10	liver/**spleen**	N/Y

*AO: Age of onset, M: male, MN: male neutered, F: female, FN: female neutered, STHS: soft tissue (localized) histiocytic sarcoma, VHS: visceral (disseminated) histiocytic sarcoma, lnn abd: abdominal lymphnodes, lnn mediast: mediastinal lymph nodes Note: For cases with VHS the site sampled for gene expressionis indicated in bold letters.*

As control tissue, normal spleen from (healthy) crossbreed dogs (n  = 6) was used as obtained at postmortem immediately following euthanasia that was not related to neoplastic, endocrine or metabolic diseases.

As a common reference pool a multitude of canine organs (testis, liver, spleen, prostate, duodenum, lung, kidney and brain) were used that had been obtained from healthy crossbreeds (n = 8) euthanized for non-metabolic, non-tumorous lethal conditions.

The procedures were approved by the local ethics committee, as required under Dutch legislation (ID 2007.III.08.110).

### RNA Isolation

Approximately 30 mg of frozen tumor was transferred to a container with 600 µl of Buffer RLT and was disrupted/lysed and homogenized using a dismembrator (Braun Biotech Int., Melsungen, Germany) for 45 s at 2200 rpm. Total RNA was isolated and treated with DNase using the RNeasy mini kit (Qiagen, The Netherlands) according to the manufacturer's protocol. Quantity and integrity were assessed with the Bioanalyzer Agilent BioAnalyzer-2100 (Bioanalyzer, Agilent Technologies, Santa Clara, CA) in combination with an RNA 6000 Pico-LabChip. The average RNA integrity number 8.5 (range: 7.2–9.8) was found to be appropriate [24]. RNA concentration was quantified using a NanoDrop ND-1000 (Isogen Life Science) spectrophotometer.

### Expression Profiling

RNA was labeled twice and hybridized against the common reference RNA on dual channel arrays. RNA concentrations were 0.6 µg/µl at a minimum amount of 3 µg per sample.

RNA amplifications and labeling were performed on an automated system (Caliper Life Sciences NV/SA, Belgium) as described [25]. Dye swap of Cy3 and Cy5 was performed to reduce dye bias. Hybridizations were done on a HS4800PRO system supplemented with QuadChambers (Tecan Benelux B.V.B.A.) using 500–1000 ng labeled cRNA per channel as described [26]. Microarrays used were Agilent Canine Gene Expression Microarrays V1 (Agilent Technologies, Belgium) representing 42,035 canine 60-mer probes in a 4×44 K layout.

Hybridized slides were scanned on an Agilent scanner (G2565BA) at 100% laser power, 30% photomultiplier tube voltage. After automated data extraction using Imagene 8.0 (BioDiscovery), printtip Loess normalization was performed [27] on mean spot-intensities. Dye-bias was corrected based on a within-set estimate as described [28].

Analyses were performed to detect common differences in gene expression between the two groups of HS and healthy spleen tissue. Data were analyzed using ANOVA [29] (R version 2.2.1/MAANOVA version 0.98–7) (http://www.r-project.org/). In a fixed effect analysis, sample, array and dye effects were modeled. *P*-values were determined by a permutation F2-test, in which residuals were permutated 5,000 times globally. Genes with *P*<0.0002 after either family wise error correction (FWER) or determination of false discovery rate (FDR) were considered significantly changed. Genes with log2-fold changes of more than 0.5 or less than −0.5 were then selected to ensure that only robust changes were considered.

The Gene Ontology (GO) database (http://www.geneontology.org/) was used to check gene molecular and biological functions of the remaining genes.

### Functional Annotation

In general, pathway analysis in dogs has its restraints because pathway identification relies heavily on existing functional annotation, which is still limited for this species. Still, pathway analysis provides an additional way to analyze expression data across species. This may shed light on common pathways important for tumor behavior and on finding new therapeutic targets. To examine whether certain pathways are over- or under-represented in the gene list, all genes significantly differentially expressed between either STHS, VHS and normal spleen, were introduced into DAVID (http://david.abcc.ncifcrf.gov/).

### Quantitative Real Time PCR

#### Gene selection

Following the outcome of the microarray expression profiling, ten genes were selected. Selection of ten genes was based on significantly differently expression, M-fold changes and potential biological function in relation with tumor development.

These genes including their optimum temperature are listed in Table 2.

**Table 2:** QPCR primers for genes of interest based on Microarray and pathway analyses.

Gene name	Accession number	Primer sequence (5'–3')	Forward/reverse	AnnealingT(°C)	Size(bp)
*PPBP*	XM_539315	ACTGTCTCTGGCATTCATCC	F	59	116
		AGGCAGATTCTCTTCCCATT	R		
*GTSF1*	XM_534784.3	GCAGAAAGAATCATCCTGATGTC	F	57	221
		GATCTTTATCCCAGTCTTCATCG	R		
*Spi-C*	XM_849901.2	AATTACCTGGCTTTCATCAACC	F	57	114
		CAGCACTGTTTATTACTGTTCTCC	R		
*VCAM1*	NM_001003298	CTACAAGTCTACATCTCACCCA	F	58	213
		TTCCAGAATCTTCCAGCCTC	R		
*ENPEP*	XM_535696.3	GCTTCCTTCTTTGAGTTCCT	F	58	266
		TTCCAAGTAAATCTGGCATCCT	R		
*LUM*	XM_539716.3	CAAGACAGAAGGATTCAAAGCA	F	55	132
		GATGACAGCCCATAAATCGG	R		
*ITGAD*	XM_843683.2	TCTTGTATTGAACTGCTCCA	F	57	261
		GTTGTAGACCTCATACTTCTCC	R		
*Col3A1*	XM_845916	ATAGAGGCTTTGATGGACGAA	F	65	132
		CCTCGCTCACCAGGAGC	R		
*MYH11*	XM_857838	GAGAGGACCAGTCCATTCTG	F	59	253
		GATGAATTTGCCAAATCGTGAG	R		
*Thy1*	XM_844606.2	CTGTGCTCAGAGACAAACTG	F	58	185
		TTAGCCAACTCAGAGAAAGTAGG	R		

*Common genes chosen for qPCR development identified using microarray as being significantly different in both disseminated, Visceral Histiocytic Sarcoma (VHS) and localized, Soft Tissue Histiocytic Sarcoma (STHS) compared to spleen. PPBP: Pro-platelet basic protein (chemokine (C-X-C motif) ligand 7), GTSF1: Gametocyte specific factor 1, SPIC: Spi-C transcription factor, VCAM1: Vascular cell adhesion molecule 1, ENPEP: Glutamyl aminopeptidase (aminopeptidase A), LUM: Lumican, ITGAD: Integrin, alpha D, Col3A1: Collagen, type III, alpha 1, MYH11: Myosin, heavy chain 11, smooth muscle, Thy-1 (CD90): Thy-1 cell surface antigen.*

### RNA Isolation and cDNA Synthesis

Tissues from all but one patient (of which the insufficient tissue remained for the qPCR experiment) were used in the microarray experiment (six spleens, six STHS and seven VHS), furthermore three additional samples (one normal spleen, one STHS and one VHS that met the inclusion criteria) were added thus creating 3 groups of seven samples for the qPCR experiment. Total RNA from these samples was isolated. After isolation, total RNA was treated with DNase using the RNeasy mini kit (Qiagen, The Netherlands) according to the manufacturer’s protocol.

Reverse transcription (RT) was performed of all 20 samples in a 80 µl reaction using 2000 ng total RNA, 16 µl iScript Reaction mix and 4 µl iScript Reverse Transcriptase (iScript cDNA Synthesis kit, Bio Rad, Veenendaal, The Netherlands). This includes a mixture of oligo-dT random hexamer primers. The mixture was incubated 5 min. at 25°C, 30 min. at 42°C and followed by 5 min. at 85°C. Minus RT controls were prepared from 500 ng of the same RNA under the same conditions, but without addition of reverse transcriptase.

### Reference Genes and Primer Development

Reference genes were selected as non-regulated reference genes for normalization based on their stable expression in canine tissue [30], .

**Figure 1:**
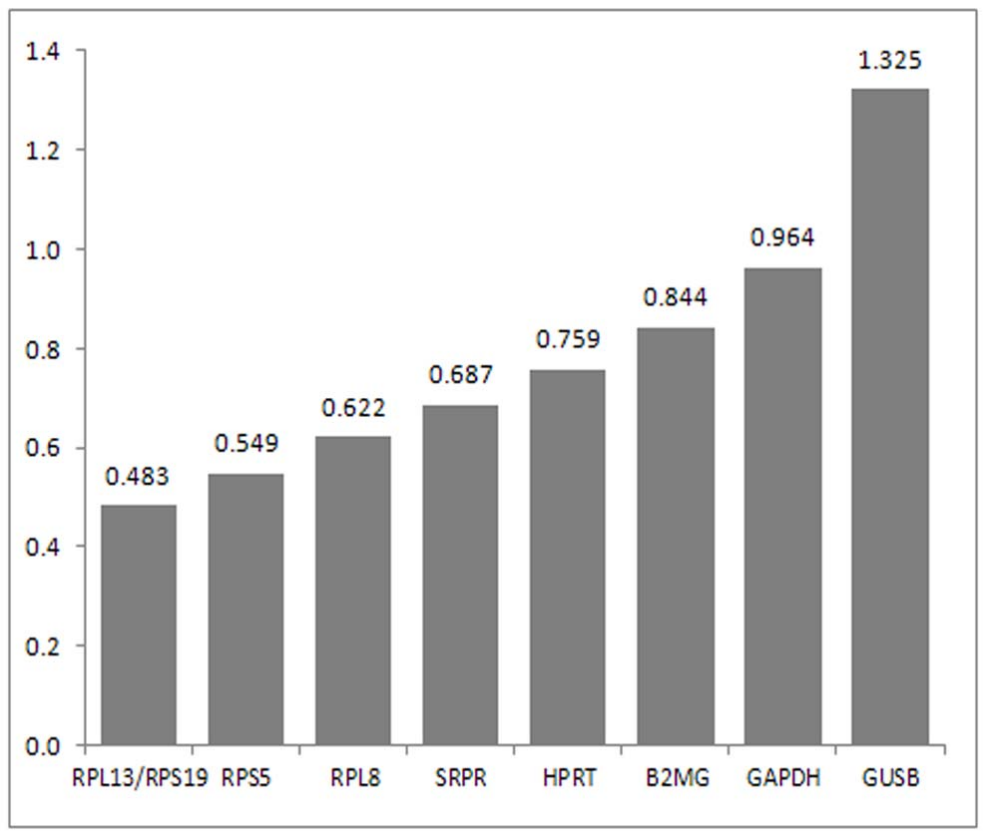
Gene stability by Genorm for all nine reference genes. Horizontal axis: Least stable genes (left) and most stable genes (right) Glyceraldehyde-3-phosphate dehydrogenase (GAPDH), β-2-Microglobulin (B2MG), Ribosomal protein S5 (RPS5), Ribosomal protein S19 (RPS19), Hypoxanthine phosphoribosyltransferase (HPRT), Ribosomalprotein L8 (RPL8), B-Glucuronidase (GUSB), Signal recognition particle receptor (SRPR), and Ribosomal protein L13 (RPL13).

Using Ensembl, through annotated transcripts, PCR primers were designed using the Perl Primer software (version 2.0.0.7) and primer3 software (version 0.4.0) according to the parameters outlined in the Bio-Rad i-cycler manual. The specificity of each primer pair was confirmed by sequencing its product and also in qPCR by checking the meltcurve and reaction efficiency. GeneNorm was used to establish expression stability [32]. Amplicon sequence-reactions were performed using BigDye v3.1 according to the manufacturer’s (Life Technologies, Bleiswijk, The Netherlands) instructions on an ABI3130×L and analyzed in Lasergene (version 9.1 DNASTAR) and confirmed the specificity of each amplicon. Using RefFinder, the stability of nine reference genes were checked. In Figure 1 they are listed according to their stability. Four of the more stable primers were chosen for further data analysis, namely *ribosomal protein S5 (RPS5), signal recognition particle receptor (SRPR), ribosomal protein L13(RPL13), hypoxanthine phosphoribosyltransferase (HPRT).*

Primers for all nine reference genes, including their optimum temperature are listed in Table 3.

**Table 3:** Reference genes primers for qPCR.

Gene name	Accession number	Primer sequence	Forward/ Reverse	Annealing T(°C)	Size (bp)
*HPRT*	AY283372	AGCTTGCTGGTGAAAAGGAC	F	56	104
		TTATAGTCAAGGGCATATCC	R		
*RPS19*	XM_533657	CCTTCCTCAAAAAGTCTGGG	F	61	95
		GTTCTCATCGTAGGGAGCAAG	R		
*RPL8*	XM_532360	CCATGAATCCTGTGGAGC	F	55	64
		GTAGAGGGTTTGCCGATG	R		
*SRPR*	XM_546411	GCTTCAGGATCTGGACTGC	F	61	81
		GTTCCCTTGGTAGCACTGG	R		
*RPL13*	AJ388525	GCCGGAAGGTTGTAGTCGT	F	61	87
		GGAGGAAGGCCAGGTAATTC	R		
*GUSB*	NM_001003191	AGACGCTTCCAAGTACCCC	F	62	103
		AGGTGTGGTGTAGAGGAGCAC	R		
*GAPDH*	NM_001003142	TGTCCCCACCCCCAATGTATC	F	58	100
		CTCCGATGCCTGCTTCACTACCTT	R		
*B2MG*	XM_535458	TCCTCATCCTCCTCGCT	F	61	85
		TTCTCTGCTGGGTGTCG	R		
*RPS5*	XM_533568	TCACTGGTGAGAACCCCCT	F	62.5	141
		CCTGATTCACACGGCGTAG	R		

*Glyceraldehyde-3-phosphate dehydrogenase (GAPDH), β-2-Microglobulin (B2MG), Ribosomal protein S5 (RPS5), Ribosomal protein S19 (RPS19), Hypoxanthine phosphoribosyltransferase (HPRT), Ribosomalprotein L8 (RPL8), B-Glucuronidase (GUSB), Signal recognition particle receptor (SRPR), and Ribosomal protein L13 (RPL13).*

### Quantative PCR

For qPCR, the Bio-Rad detection system (Bio-Rad.) with SYBR green fluorophore was used. Reactions were performed in a total volume of 25 µl containing 12.5 µl of 2×SYBR green super mixes (Bio-Rad Laboratories Ltd.), 1 µl of each primer at 400 nM concentration, 0.8 µl of cDNA and 9.7 µl Rnase and Dnase free water as previously described [33], [34]. Q-PCR reactions for each primer set were optimized by performing reactions under a gradient of annealing temperature using three serial 16×dilutions of pooled cDNA from all tissue samples. Cycling conditions were as follows: Denaturation (95°C for 5 min.), amplification cycle repeated 45 times (95°C for 10 sec, 30 sec at the primer specific annealing temperature (Table 2) and 30 sec at 72°C. The last step, 30 sec at 72°C was omitted when the annealing temperature was higher than 58°C. A melting curve analysis was performed following every run to ensure a single amplified product for every reaction. All reactions were performed in duplicate. The reference standard dilution series was repeated on every plate. Duplicate negative controls, both a minus RT and a water control, were run with every experimental plate to assess the specificity and to identify any potential contamination.

Data analysis was performed with IQ5 Real-Time PCR detection system software (BioRad). Expression levels were normalized using the average relative amount of the reference genes. Log-values of normalized relative expression were used to obtain normal distribution.

A Wilcoxon rank sum test was performed to determine the significance of differential gene expression. All results were Bonferroni corrected.

## Results and Discussion

Differentiation between STHS and VHS was based on physical examination and radiographic (thorax)/ultrasound examination (thorax/abdomen). All dogs with visceral organ involvement were euthanized followed by immediate autopsy. Histomorphology and immunohistochemical staining for CD18 confirmed the suspected histiocytic origin of tumors studied.

The Microarray enabled analysis of the expression of 42,034 features. Since only 21,682 (51% ) were annotated (CanFam 2.0), it is possible that important genes are missed. When comparing VHS and spleen, 4,235 features were significantly differentially expressed. When only looking at 4-foldchanges or larger, 352 features remained. When comparing STHS with spleen, 5,779 features were significantly differentially expressed. In this comparison, when only looking at 4-foldchanges or larger, 437 features remain.

Of the total of altered genes, 3,394 features were significantly differentially expressed in both forms of HS versus normal spleen, and 319 features remained when only 4-foldchanges or larger are taking into account.

**Figure 2:**
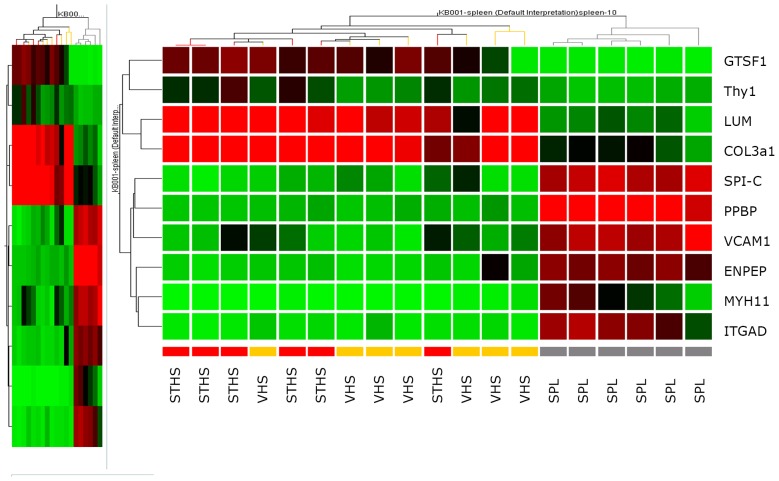
Microarray-based heatmap of the ten genes chosen for qPCR. PPBP: Pro-platelet basic protein (chemokine (C-X-C motif) ligand 7), GTSF1: Gametocyte specific factor 1, SPIC: Spi-C transcription factor, VCAM1: Vascular cell adhesion molecule 1, ENPEP: Glutamyl aminopeptidase (aminopeptidase A), LUM: Lumican, ITGAD: Integrin, alpha D, Col3A1: Collagen, type III, alpha 1, MYH11: Myosin, heavy chain 11, smooth muscle, Thy-1 (CD90): Thy-1 cell surface antigen.

Figure 2 visualizes the heatmap of the ten genes that were chosen for qPCR confirmation.

In order to improve knowledge on the genetic basis of HS and to best exploit logistic and financial resources, we chose to examine a selection of the significantly altered genes found in the microarray experiment for confirmation by qPCR. Selection of genes was based on the statistical significance of their differential expression and their potential involvement in tumor development.

Since it is not possible to obtain pure samples of sedentary, non-tumorous histiocytes for expression profiling, spleen tissue was chosen as the normal equivalent of HS. HS arises from interstitial DC, and hence emanate from lymphoid domains when these arise from lymphoid organs such as the spleen [6], choosing spleen as a control tissue is a logical choice.

As a result from the qPCR experiment, significantly altered expression was confirmed for nine of the ten genes analyzed. *PPBP, SpiC, VCAM1, ENPEP* and *ITGAD* were downregulated and *GTSF1, LUM*, *Thy1* and *Col3a1* were upregulated in both STHS and VHS compared to normal spleen. Table 4 shows the adjusted *p*-values and Fold Change.

**Table 4:** Genes identified as potential interesting using gene profiling in both VHS and STHS compared to spleen.

Gene name	qPCR:Adj *p*-values VHS vs Spleen	qPCR:Adj *p*-values STHS vs Spleen	qPCR:Up/Down regulated versus spleen	Spleen versus VHS resp STHS; Fold Change
*PPBP*	7.6×10^−8^	7.23×10^−9^	DOWN	−360×, −1008×
*GTSF1*	2.9×10^−4^	5.25×10^−5^	UP	1000×, 1000×
*Spi-C*	2.0×10^−3^	2.4×10^−4^	DOWN	−12×, −51×
*VCAM1*	0.011478	1.1 10^−4^	DOWN	−10×, −6×
*ENPEP*	8.4×10^−4^	2.0×10^−8^	DOWN	−324×, −1472×
*LUM*	3.4×10^−3^	5.43×10^−7^	UP	88×, 48×
*ITGAD(CD11d)*	8.5×10^−3^	1.6×10^−3^	DOWN	−4×, −253×
*Col3a1*	7.0×10^−3^	0.010	UP	23×, 18×
*Thy1 (CD90)*	0.024	4.19E-05	UP	3×, 6×

*PPBP: Pro-platelet basic protein (chemokine (C-X-C motif) ligand 7), GTSF1: Gametocyte specific factor 1, SPI-C: Spi-C transcription factor, VCAM1: Vascular cell adhesion molecule 1, ENPEP: Glutamyl aminopeptidase (aminopeptidase A), LUM: Lumican, ITGAD: Integrin, alpha D, Col3A1: Collagen, type III, alpha 1, Thy-1 (CD90): Thy-1 cell surface antigen.*

The use of spleen as the healthy equivalent of HS does raise some concern as to how observed differences in *Spi-C* and *VCAM1* gene expression between tumors and healthy spleen must be explained. It is possible that some of these expression differences are based on differences in tissue-origin rather than on actual tumor development. *Spi-C* plays a critical role in the development of splenic iron homeostasis. It is highly expressed in red pulp macrophages, but not monocytes, dendritic cells or other tissue macrophages. *Spi-C* is therefore highly expressed in spleen [35], and could thus lead to cause a seemingly relative down-regulation in HS tissues. *Spi-C* is also known to regulate *VCAM1* expression [35]. This gene is thought to be involved in angiogenesis and is induced by cytokines on endothelial cells [36]. Since spleen tissue contains abundant endothelium, this could cause the relative high expression of *VCAM1* in the spleen. *ITGAD* (also known as *CD11d*) is a receptor for *VCAM1*. In our study, we detected a lowered expression of *CD11d* in HS as compared to normal spleen.

Staining by immunohistochemistry for the presence of CD11d in both STHS and VHS was found negative in a first study by Moore et al, including16 splenic HS [6] and this absence of the CD11d protein in HS was seen as one of the phenotypically characteristics of a myeloid dendritic antigen-presenting cell lineage, making many HS to be likely myeloid dendritic cell sarcomas [6]. In contrast, a more recent study in Flatcoated Retrievers found the majority (12/20) of splenic HS positive for CD11d [8] and was interpreted by the investigators as marker of a likely red-pulp macrophagocytic origin of these splenic HS. A similar rate of CD11d positivity was noticed by another study by Moore et al examining hemophagocytic HS in spleen in a series of dogs from 6 breeds [7]. CD11d proteins appear to be strongly expressed only on mature granulocytes, monocytes, and certain lymphocytes, but not significantly on myeloid committed precursor cells [37] and hence, the low expression in many HS is no surprise as is its positivity in hemophagocytic HS. Still, the finding of positive expression in splenic HS by immunohistochemistry [8] is at variance with earlier studies [6] and was interpreted as marker of a likely red-pulp macrophagocytic origin of these splenic HS. In our study, we detected a lowered expression of *CD11d* in HS as compared to normal spleen.

The down-regulation of *PPBP* as was found in this study, has also been reported to play a role in the development of myelomas [38] as well as pancreatic cancers [39], in the latter report the PPBP (CXCL7) plasma-level was even postulated to be an interesting biomarker for early detection [39]. The close relation in the origin of myeloma and histiocytic sarcoma, both stemming from lineages of hematoproliferative compartments makes this downregulation of *PPBP* an intriguing observation worthy of further pursuit.

Expression and overexpression of *LUM* has been observed in various types of cancer cells (colorectal, pancreatic, and breast cancer, melanoma, neuroendocrine cell tumors) - with contrasting findings on the relation with type of growth and/or tumor progression or metastasis [40]–[46] - and in activated synoviocytes from rheumatoid arthritis [47]. The function of lumican - a member of the family of small leucine rich proteoglycans - in the organization of the extracellular matrix composition as well as in migration and proliferation- in relation to the observed overexpression in HS warrants further investigations, including the site of overexpression (tumor cell or tumor-associated fibroblasts) [48].

In humans, an up regulation of *GTSF1* is already known in the occurrence of mycosis fungoides, which is the most common type of primary cutaneous T-cell lymphoma, in which *GTSF1* was proposed as a gene for which expression appears to be restricted to mycosis fungoides tumor stage and that might even serve as diagnostic (bio)marker [49].

*ENPEP* probably plays a role in regulating growth and differentiation of early B-lineage cells (http://www.wikigenes.org/e/gene/e/13809.html) and down regulation may thus also be involved in HS development.

Finally, *Col3a1* also has been shown to be overexpressed in other types of tumors, such as malignant mesothelioma [50], as well as in human sarcoma xenotransplants [51]. Its overexpression could have a significant influence upon extracellular matrix composition [52].

*Thy1* (*CD90*) is an important marker of many types of stem cells [53], including mesenchymal stem cells [54]. *CD90* has already been identified as a candidate marker for cancer stem cells in primary high-grade gliomas using tissue microarrays [55], [56]. For human hepatocellular carcinoma, *CD90* has even been shown to provide a clinical prognostic marker [53]. Our observation of overexpression of *CD90* in HS might herald stem cell characteristics of the type of cancer.

For technical reasons, no qPCR data could be obtained for *Myh11.*

As a result of the pathway analyses in DAVID, 24 pathways were significantly involved in the development of HS (*P*<0.05). Most were involved in the DNA repair and replication process. The biological functions of ten of these pathways, amongst which the P53 signaling pathway was one of the most relevant, are listed in Table 5.

**Table 5:** Ten of the most significant pathways that resulted from the pathway analyses in DAVID.

**Category**	**Term**	**No of genes**	**P-value**
KEGG_PATHWAY	cfa04142:Lysosome	25	1.62E-05
KEGG_PATHWAY	cfa04110:Cell cycle	26	3.44E-05
CHROMOSOME	3	59	4.09E-05
CHROMOSOME	5	102	4.92E-05
CYTOBAND	3	59	5.78E-05
CYTOBAND	5	102	7.71E-05
KEGG_PATHWAY	cfa03030:DNAreplication	11	4.61E-04
KEGG_PATHWAY	cfa03050:Proteasome	12	0.0016
KEGG_PATHWAY	cfa04115:p53signaling pathway	13	0.0076
KEGG_PATHWAY	cfa03430: DNAmismatch repair	7	0.0076

Our observations provide evidence of an association between altered expression of nine genes (*PPBP, SpiC, VCAM1, ENPEP, ITGAD, GTSF1, COL3A1, CD90* and *LUM*) and development of HS in the Flatcoated retriever dog, irrespective of the disseminated or localized form.

Based upon a fundamental and evolutionarily conserved association between cytogenetic abnormalities and tumor phenotype in different species [10], [57] these genes may be of major interest in the study of histiocytic malignancies in the human as well. There exists a great difference in incidence of histiocytic malignancies between this specific dog breed as compared to the human. This implies a genetic make-up predisposing the Flatcoated retriever (and the Bernese Mountain Dog); which is uncommon in many other dog breeds and humans alike. For the Bernese Mountain Dog, a first genetic locus has been identified. Our group takes part in a study aiming to identify predisposing genes in the Flat Coated Retriever in the hope, that these findings may provide clues for the related cancer in the human.

The current study provides the most comprehensive database of genome alterations in histiocytic malignancies to date, revealing genes and signaling pathways not previously associated with this disease. Although mRNA levels do not necessary reflect differences in protein levels, it is very well conceivable that the large difference in mRNA levels of specific genes will result in quantitative differences in protein expression. Lack of verified and specific antibodies for all nine gene products of interest let us to restrain this expression profiles to mRNA levels only. The study of Hedan [10] was able to locate recurrent genomic imbalances using CGH. As indicated in Table 3 of their publication [10], 808 genes found to be located in their regions of interest. This covers about 4% of the total number of genes. In the Agilent Canine Gene Expression Microarrays V1 that were used in our study, 430 of these 808 genes were annotated. Three of these annotated genes; ENSCAFG00000007012 (*SPIC*), ENSCAFG00000001046 (*DESI1*) and ENSCAFG00000006138 (*LUM*) were found to be commonly involved in our study as well as in Hedans study. In our study, eventually two of these genes, *SPIC* and *LUM*, were chosen for qPCR confirmation. We were indeed able to identify the significantly altered expression of these two genes.

## Conclusion

This is the first study to compare gene expression in HS (both STHS and VHS) and normal spleen using both traditional fold change analysis as well as disease-based pathway analyses using DAVID.

This study provides evidence for involvement of several genes in HS, irrespective of the form of manifestation, some of which are also related with to other cancers. On the basis of quantitative differences in expression, we consider *PPBP, SpiC, VCAM1, ENPEP, ITGAD* (down-regulated), and *GTSF1, Col3a1, CD90* and *LUM* (up-regulated) to be associated with the HS genotype.

Extrapolation of this data to human samples may help to further our understanding of the propagation and oncogenesis of histiocytic cells. Eventually, this will contribute to the development of effective therapeutic modalities for both species.
